# Supplementary figures and images for: Serratia sp. traits distinguish the lung microbiome of patients with tuberculosis and non-tuberculous mycobacterial lung diseases
Source: PLoS One. 2025 Jun 26;20(6):e0325362. doi: 10.1371/journal.pone.0325362 (PMC12200645; doi:10.1371/journal.pone.0325362)

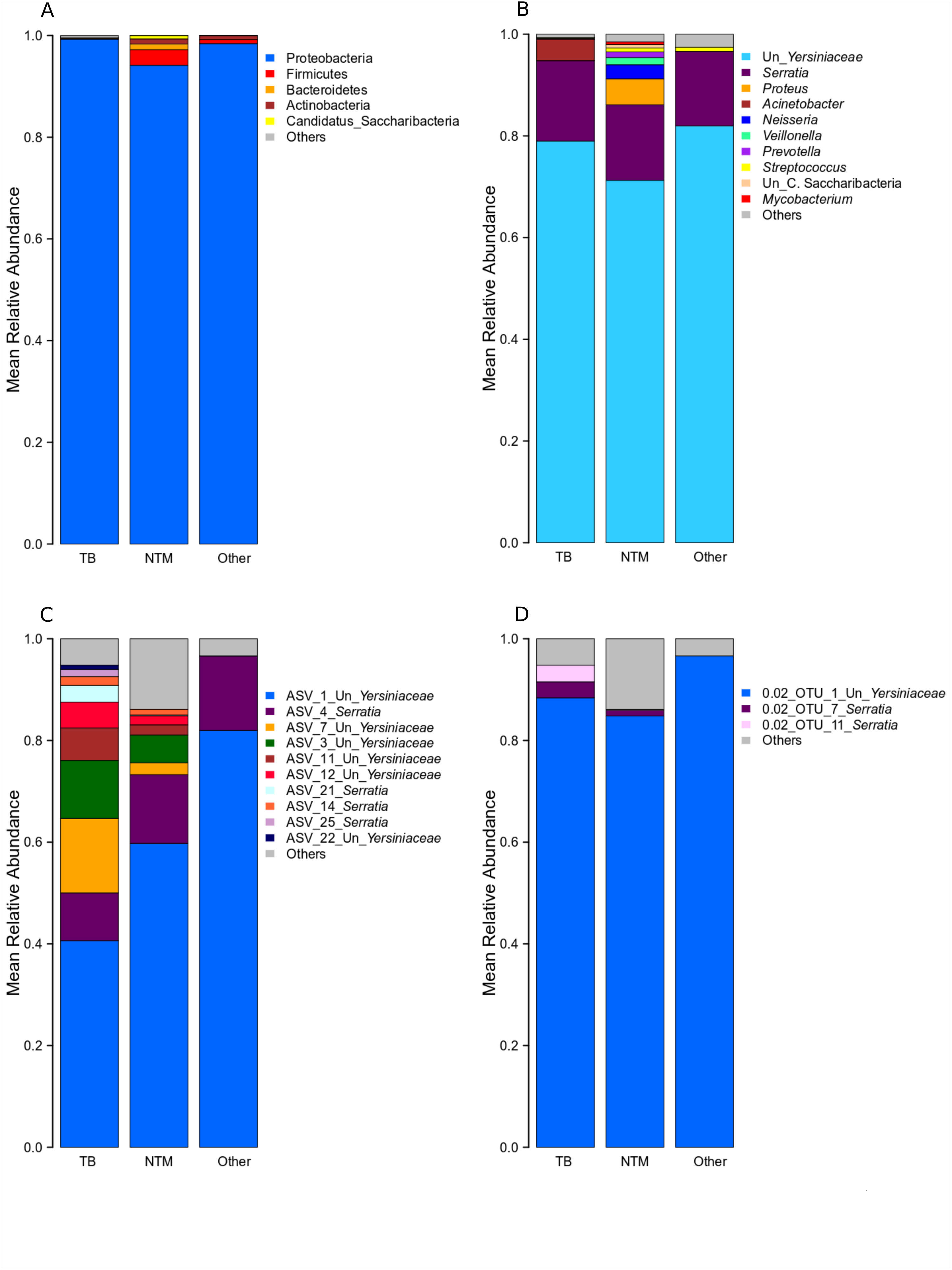

Supplement: S1 Fig — A. Phyla, B. Genera, C. ASVs, D. 98% OTUs. Un: unclassified. (TIF) [file pone.0325362.s001.tif]

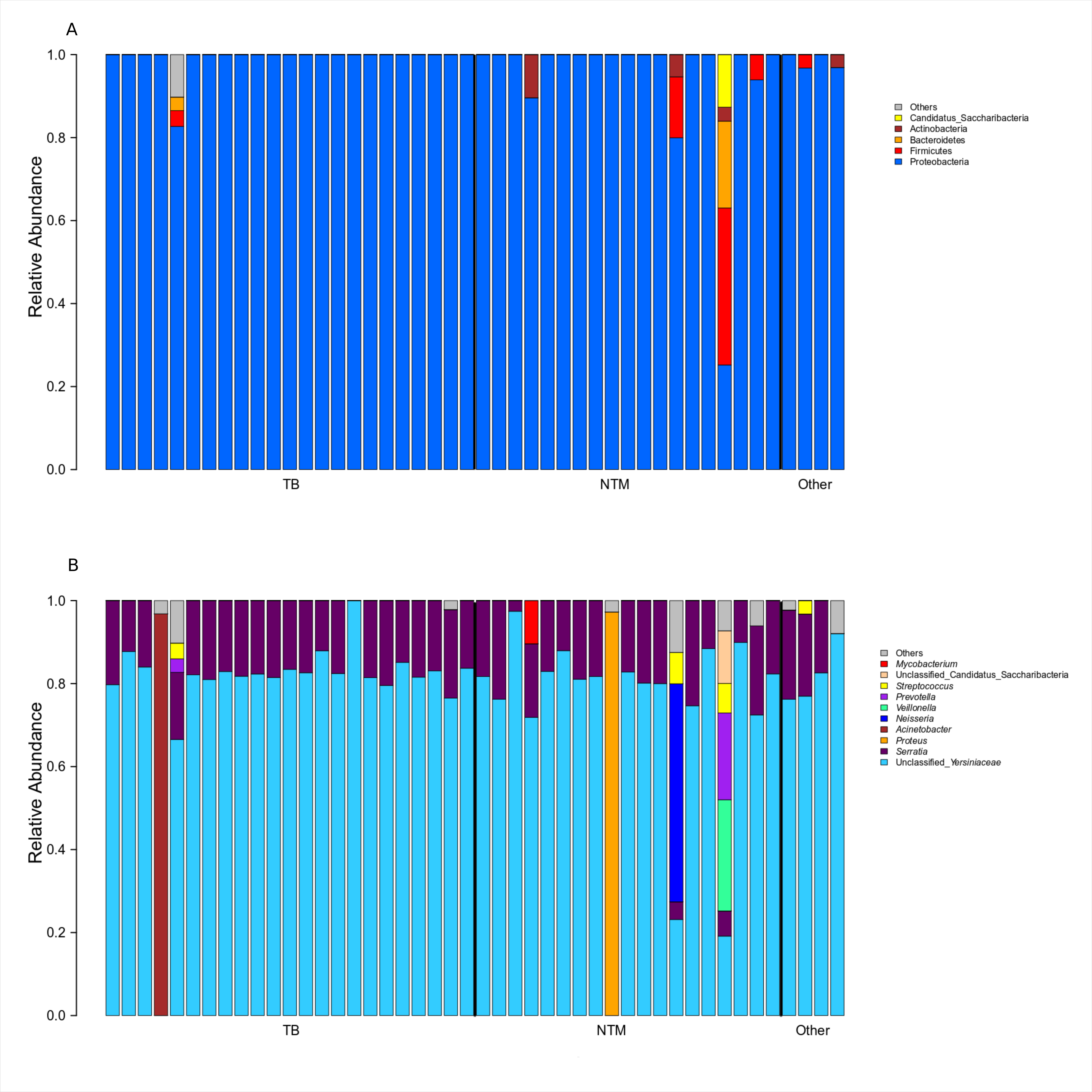

Supplement: S2 Fig — A. Phyla, B. Genera. (TIF) [file pone.0325362.s002.tif]

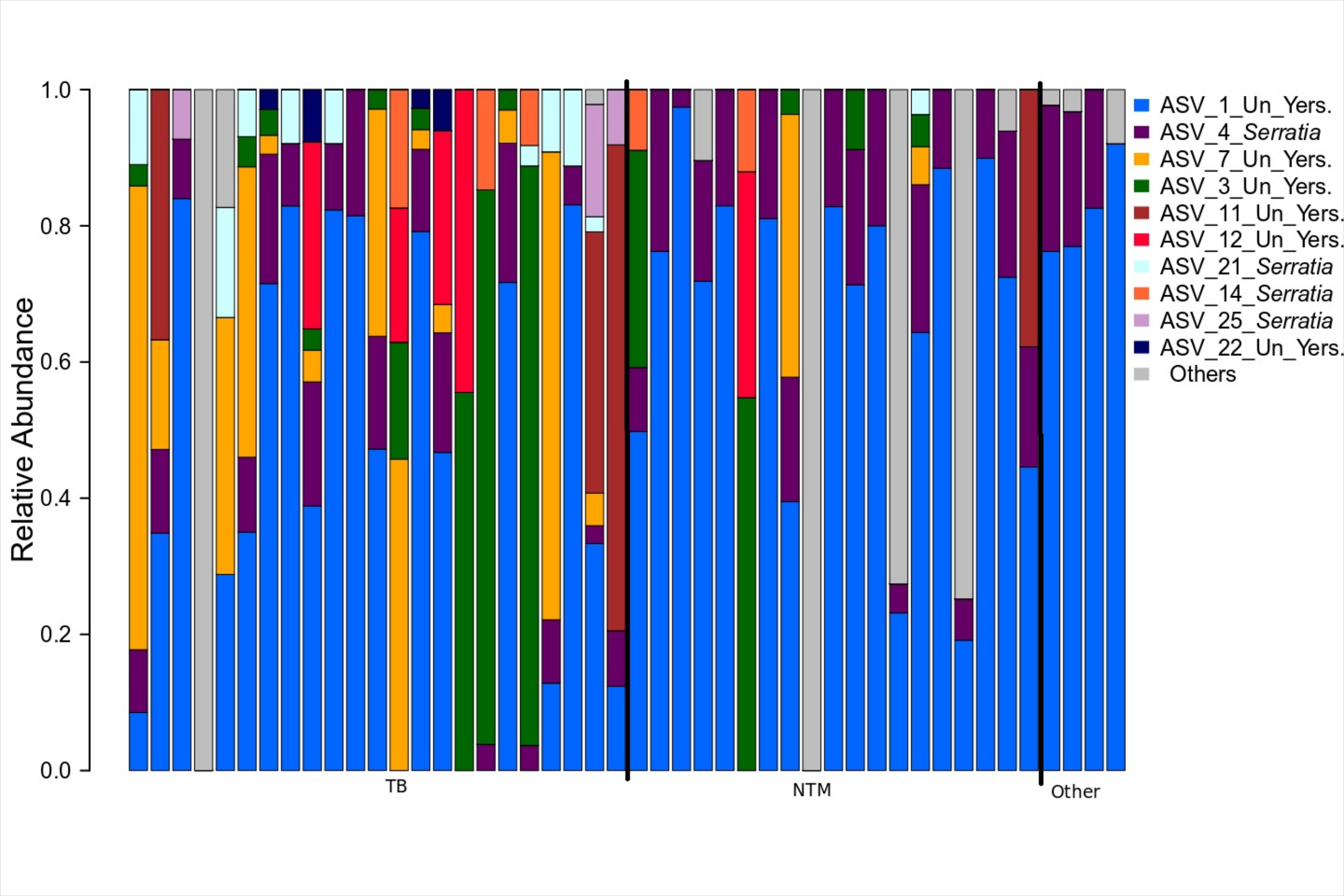

Supplement: S3 Fig — (TIF) [file pone.0325362.s003.tif]

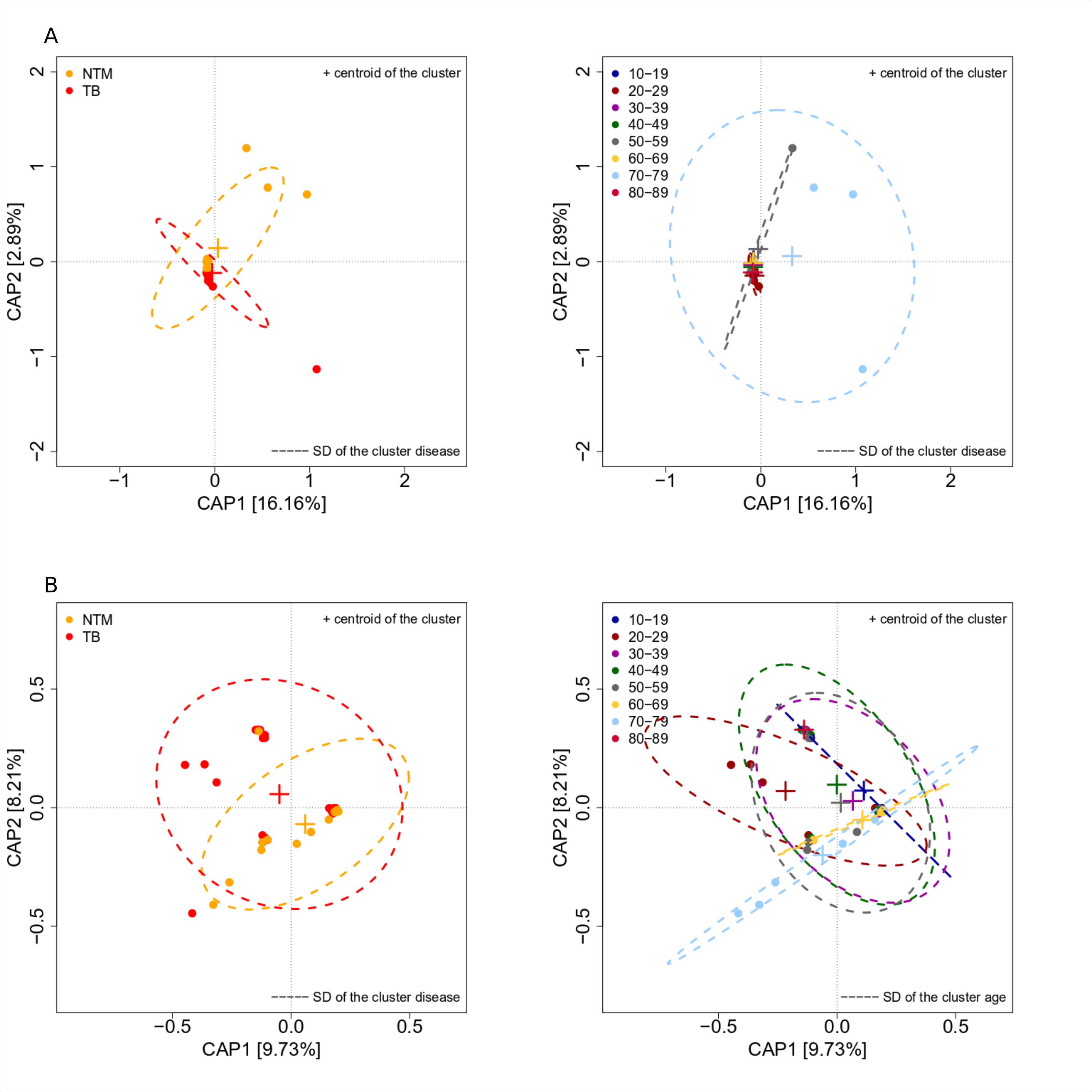

Supplement: S4 Fig — (TIF) [file pone.0325362.s004.tif]

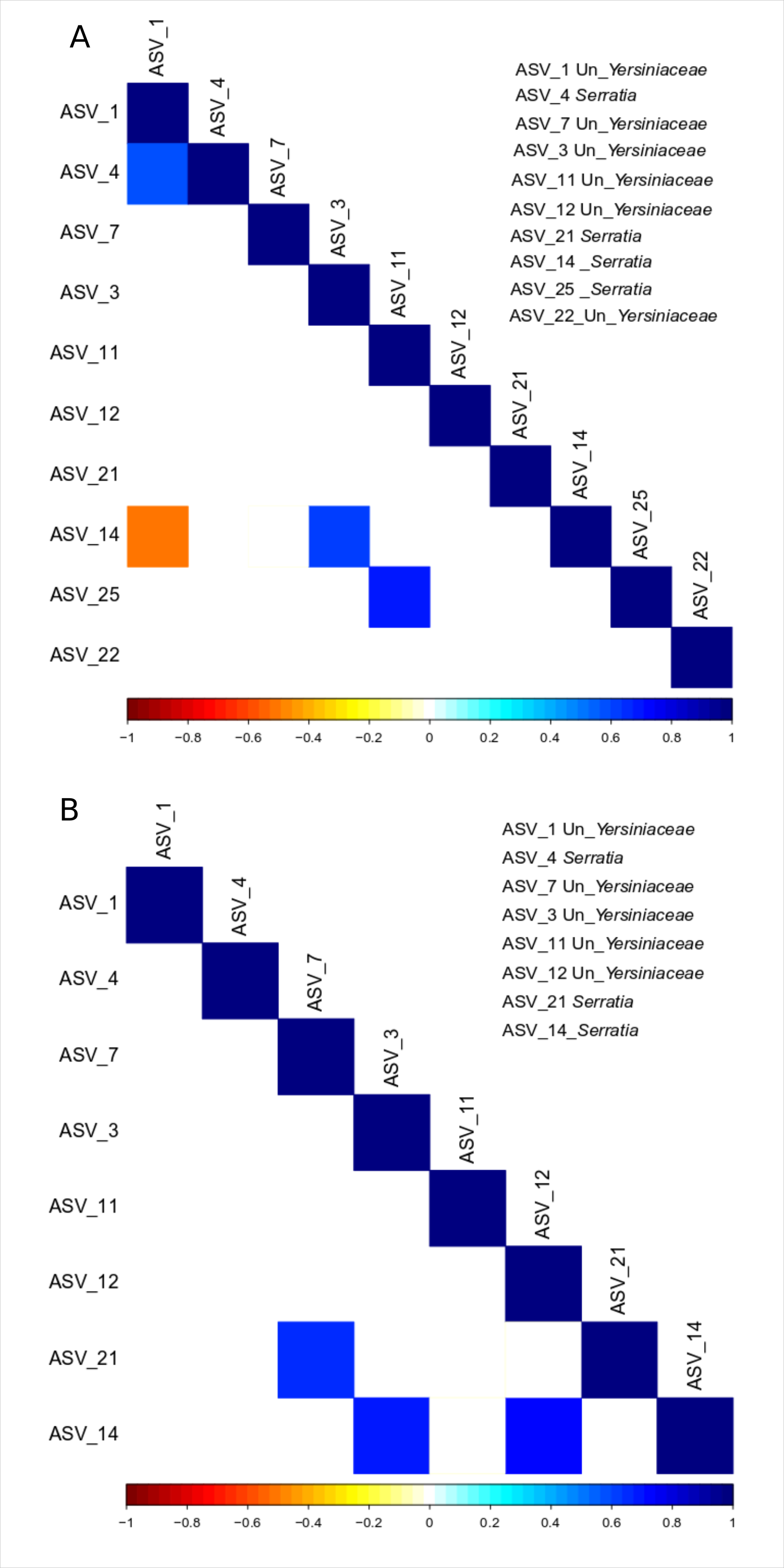

Supplement: S5 Fig — A. TB, B. NTM. Only significant correlations after p values correction are shown. Un: Unclassified. (TIF) [file pone.0325362.s005.tif]

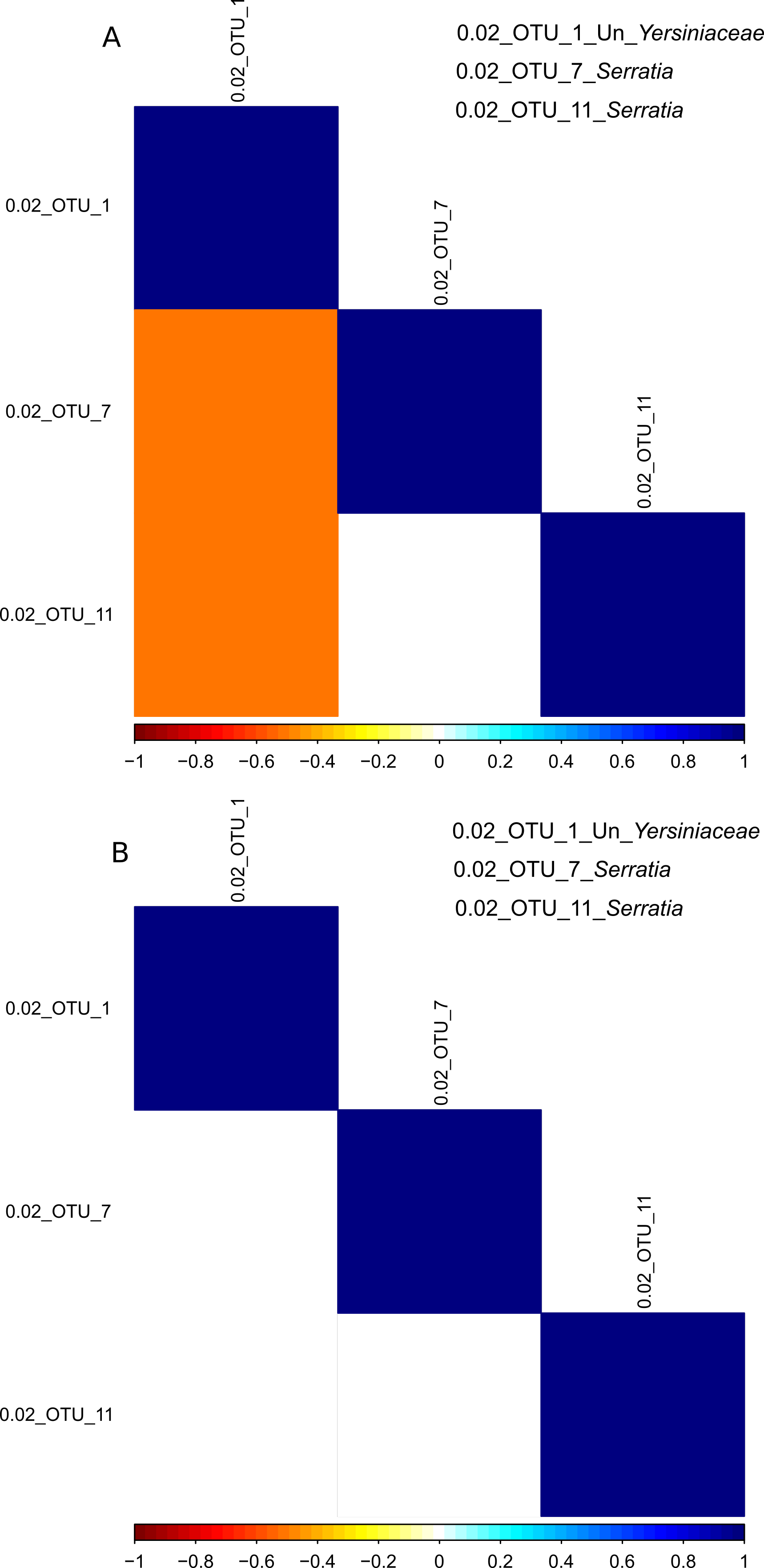

Supplement: S6 Fig — A. TB, B. NTM. Only significant correlations after p values correction are shown. Un: Unclassified. (TIF) [file pone.0325362.s006.tif]

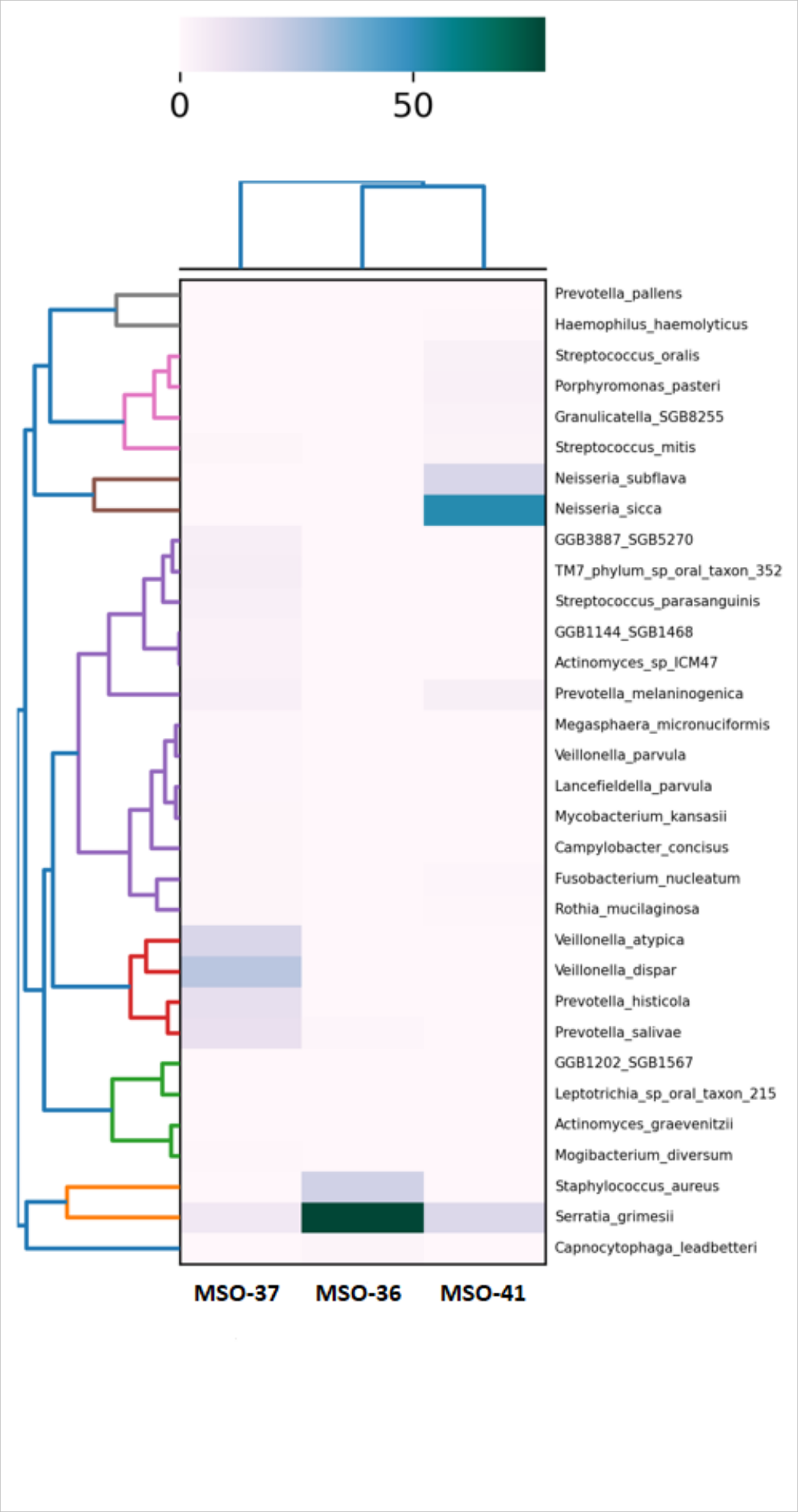

Supplement: S7 Fig — Only species with a relative abundance >0.1% are shown, with Serratia grimesii occurring in all 3 BALF specimens (from left to right, 7%, 78%, and 16%, respectively). (TIF) [file pone.0325362.s007.tif]
