# Supplementary material for: Serratia sp. traits distinguish the lung microbiome of patients with tuberculosis and non-tuberculous mycobacterial lung diseases
Source: PLoS One. 2025 Jun 26;20(6):e0325362. doi: 10.1371/journal.pone.0325362 (PMC12200645; doi:10.1371/journal.pone.0325362)
Supplement: Attachment — Submitted filename: revision_letter_2025-04-24.docx [file pone.0325362.s013.docx]

To the Editorial Board of PlosOne

**Dr. Matthias Merker**

**Associate Professor**

**Head, Evolution of the Resistome**

**Research Center Borstel**

**Parkallee 1-40**

**23845 Borstel, Germany**

**E-Mail: mmerker@fz-borstel.de**

**Phone: 0049-4537-1887570**

www.fz-borstel.de

Subject: PONE-D-25-06704

Borstel, April 25^th^, 2025

Dear Dr. Selvakumar Subbian,

We thank you and the peer reviewers for the encouraging responses and suggested improvements to our manuscript entitled:

**Serratia sp. traits distinguish the lung microbiome of patients with tuberculosis and non-tuberculous mycobacterial lung diseases**

Following the provided guidance, we have revised the manuscript accordingly, and provide a point-by-point response below (authors’ comments in blue).

**Reviewer #1:** This manuscript investigates the lung microbiome composition in patients with tuberculosis (TB) and non-tuberculous mycobacterial lung diseases (NTM-LD) using 16S rRNA amplicon sequencing and exploratory whole-metagenome sequencing (WMS). The study presents an interesting analysis of bacterial taxa associated with disease states, particularly highlighting Serratia and Yersiniaceae as potential key players. While the research contributes to our understanding of the lung microbiome in TB and NTM-LD patients, addressing several key concerns regarding statistical rigor, interpretation of biological findings, and contextualization within prior literature would greatly enhance the clarity and impact of the study.

**Reviewer comments:**

1. Statistical Support
The study employs appropriate microbiome analysis methods, including diversity indices, indicator species analysis, and PERMANOVA (adonis test). However, certain statistical limitations may affect the reliability of the conclusions.

- Sample size
The study analyzes 46 BALF samples, comprising 23 from TB patients, 19 from NTM-LD patients, and only 4 from individuals with non-infectious inflammatory lung diseases. Expanding the control group, particularly by including a larger cohort or, if feasible, healthy individuals, would help better differentiate disease-specific microbiome variations from general lung pathology. Strengthening the control group would improve the robustness of comparisons between infectious and non-infectious conditions and provide clearer insights into the unique microbial signatures of TB and NTM-LD.

Thank you for your comment. Though we agree that the sample size of the non-infectious patient group is low, we argue the following:

i) BALF specimens from healthy controls are not available for Ethics reasons: since the bronchoscopy is a very invasive sampling technique, the healthy controls group are usually patients of non-infectious lung diseases.
ii) Here we carefully selected from the available cohort, BALF specimens which were adequately stored, and had sufficient patient records/metadata.
iii) We plan to present the findings of this study to start a bigger recruiting effort to follow-up on these first results.

-Consideration of Confounding Variables and possible limitations
Incorporating potential confounding factors would enhance the interpretability of microbiome differences across patient groups. Key factors that could influence microbial community composition include:

Antibiotic exposure (both past and present), which may significantly alter microbiome diversity; comorbidities (e.g., HIV, hepatitis, COPD, smoking history), which could contribute to microbiome variability; immune status, as host immune responses can shape microbial colonization patterns; while age and sex distributions are acknowledged, further modeling their impact in statistical analyses or discussing their potential influence as a study limitation would add important context to the results and provide a foundation for future TB-microbiome research.

Thank you for your suggestion. We fully agree that potential confounding factors need to be accounted for to disentangle the true impact of the disease on the microbiome composition. Indeed, certain patients had further pathologies including COPD or bronchiectasis among NTM patients, and HIV or Hepatitis B among TB patients. However, the sample size among each morbidity/secondary diagnosis was 1 or 2. Given our overall small sample sizes (NTM n=19, TB, n=23) we reasoned we did not have enough statistical power to reliably test the effect of these secondary diagnosis, and so their potential unique influence would remain in the fraction of unexplained observed variability. We did however consider all the patient records we were able to collect:

Present antibiotic treatment: Through the questionnaire, we established that the patients have not been taking anti-TB treatments at the time of the bronchoscopy.

Past antibiotic treatment: based on the questionnaire, and information handed in by the patients, we had only one TB patient who has been taking anti-TB drugs a decade before sampling date: n=1 gives no robustness for statistical analyses.

Further medication: we collected medication data, and tested use of steroids medication.

Overall on the lung microbiome, we tested the following variables disease group, age group, sex, smoking status, steroid medication, and sampling year. When variation was observed, and there was enough sample size for statistical testing, the variable was included in the final statistical model: Main text lines 100-101, and supplementary material 277-288.

Finally, we do mention the incomplete prior medical history as a limitation: lines 342-344.

While age and sex distributions are acknowledged, further modeling their impact in statistical analyses could clarify their influence on microbiome diversity and structure. Addressing these aspects, either through statistical adjustments or discussion of their potential effects and therefore limitations of the study, would add important context to the results and pave the way to further analysis in TB-microbiome.

We agree that the impact of sex, and age needs to be further evaluated to clarify their influence on the microbiome diversity, and structure. This is why i) we assessed along with disease group, the fractions of variance explained by age, sex, smoking status, steroid medication, and sampling year on the structure of the microbiome (namely Bray-Curtis, and Jaccard indices) using the non-parametric ANOVA “adonis”, and constrained principal coordinates analyses “capscale”: Lines 97-100 (Methods), lines 196-216 (Results); lines 257-266 (Supplementary Material). These analyses indicated that age marginally impacted Bray-Curtis index, while age influence on the presence-absence Jaccard index was not significant.

Furthermore, to validate the indicator analysis results which identified ASV_7, ASV_21, and OTU_11_0.02 as indicator of TB disease state. For each indicator taxon, we constructed a linear model (lm) in R with relative abundances a response variable, and disease group, sex, age category, and sampling year as explanatory variables lines 227-288 (Supplementary Material).

We found that disease state, and age significantly impacted ASV_7 whereas only disease state significantly influenced ASV_21, and OTU_11, and age, and sampling year had no significant effect on these taxa.

- Interpretation of Microbiome Associations
The observed differences in amplicon sequence variant (ASV) abundance between TB and NTM-LD patients offer valuable insights into microbiome variability. However, additional analyses would help determine whether these differences are functionally relevant, rather than reflective of natural variation or potential contamination.

Thank you for your comments. To determine whether these differences are:

i) functionally relevant: This point needs experiments in a subsequent study, to determine how these differentially abundant ASVs alter the progression of the infection, and response to the therapy.
ii) rather than reflective of natural variation: We thoroughly checked the literature, and found that *Yersiniaceae*/*Serratia sp.* are hardly detectable in healthy controls.
iii) or potential contamination: we performed numerous analyses to assess the likelihood of *Serratia* and *Yersiniaceae* taxa of being environmental contaminants, and concluded that this likelihood was very limited.

Since ASV variability alone does not establish a causal or mechanistic role in disease, integrating complementary approaches—such as functional profiling, metagenomic pathway analysis, or validation in independent cohorts—could provide a more comprehensive understanding of the potential role of Serratia and Yersiniaceae in TB and NTM-LD.

Thank you for your comment. The main scope of this study is descriptive, as a basis for future investigations to elucidate the functional mechanisms of ASVs variability in further cohorts. We did perform explorative metagenomic analysis to fine scale the ASV taxonomy. A future investigation where BALF material will be sampled also for culturomics purposes is appropriate for elucidating the causal or mechanistic role of *Serratia* and *Yersiniaceae* taxa in the disease, using *in vivo* and *in vitro* models.

Additionally, discussing alternative explanations for microbial shifts (e.g., environmental influences) would help ensure that conclusions are well-supported.

Thank you for your comment. In the discussion, we do address explanations for the microbiome disparities across patient groups; we proposed impaired colonization resistance to explain the higher diversity in TB patient lines 265-267 (Discussion). In addition, we suggested further mechanisms to potentially explain the microbiome heterogeneity in TB including the exact infectious strain(s), duration of the infection, physiopathology of the lungs, and now added the damaged gut-lung barriers since there more evidence the gut microbiome also influences lungs health line 394 (Discussion).

2. Interpretation of Biological Findings
The study provides an intriguing analysis of Serratia and Yersiniaceae differences between TB and NTM-LD patients. Strengthening the discussion around the biological significance of these findings would further enhance the impact of the study. Clarifying whether these differences reflect disease-specific microbiome shifts, opportunistic colonization, or external factors would help frame the results in a broader microbiological and clinical context.

Thank you for your suggestion.

i) differences reflect disease-specific microbiome shifts🡺 Our analyses support this claim since we identified indicator taxa for TB infection.
ii) opportunistic colonization 🡺 we propose impaired colonization resistance as a potential explanation for the higher taxa diversity within TB patients.
iii) or external factors would help frame the results in a broader microbiological and clinical context🡺 We assessed the effects of the variables collected including sampling year, and steroid medication.

Overall our results revealed a distinct microbiome structure across TB, and NTM patients, with TB harboring a higher microbial heterogeneity compared to NTM. We found a significant influence of disease on microbiome structure which explained between 5.7%, and 7.2% of the variance in diversity, and between 14%, and 16% of variance in abundances of single taxa.

Previous studies have indeed revealed *Serratia* to dominate the lungs of patients of diverse pathologies such as COPD, pneumonia, cancer, and TB. Of note, Hu et al., 2020 found *Serratia* OTUs which highly dominated MTB+ patients, decreased after negative conversion following anti-TB therapy. These studies indicate that *Serratia* traits colonize compromised lungs, and *Serratia* is likely an opportunistic pathogen.

Besides, we also revealed *Serratia* dominated all patient groups, yet with disease-specific aspects: TB patients harbored a more heterogenous, and diversified microbiome structure driven by distinct *Serratia* traits, and we identified ASVs which were significantly associated with TB. Our results, along with the recent *in vitro* assays on the growth-promoting effect *Serratia* *marcescens* towards *Mycobacterium tuberculosis* suggest disease-specific colonization patterns of *Serratia* in the lungs.

Overall, previous studies, and our results suggest that *Serratia* colonization of the infected lungs is likely opportunistic, and the specificity of the traits, and their interactions within the lung microbiome is likely disease-specific.

We included this conclusion in the manuscript, discussion section, lines 268-279

Hu Y, Kang Y, Liu X, et al. Distinct lung microbial community states in patients with pulmonary tuberculosis. 2020; : 63(10):1522-1533.

2.1 Potential Contamination in Low-Biomass Samples
Since Serratia and Yersiniaceae are known to be prevalent in laboratory and hospital environments, it would be helpful for the manuscript to briefly discuss measures taken to rule out contamination and further contextualize the robustness of the findings. While the study employs a rigorous decontamination strategy, low-biomass samples like BALF are particularly susceptible to external microbial influences, which could have an impact on microbial abundance estimations. (doi: 10.1186/s40168-016-0172-3; 10.1186/s41479-018-0051-8).

Thank you for your comment. We explicitly, and rigorously addressed this point, applied the recommended decontamination strategies, and used a diverse panel of positive, and negative controls to detect, and exclude contaminant, and spurious ASVs from BALF samples, and also excluded BALF samples which we could not confidently decontaminate. Our decontamination scheme is described in the manuscript lines 330-337 (strengths, and limitations), and in details in the supplementary material lines 113-179.

In brief, our strategy for the detection of contaminant, and spurious ASVs was based on:

- DNA extraction negative controls, and PCR reagents negative controls for each primer combination: DNA extraction controls were included in the final library if amplification was detected, whereas PCR regents’ controls were required to be amplification-free.
- We applied the frequency method of the decontam algorithm to detect contaminant sequences which negatively correlated with samples biomass, with selected, and adjusted parameters (probability thresholds) based on the positive controls included in the dataset: microbial community standards, pure cultures, and technical replicates.
- Positive controls, and technical replicates: Following the recommendations of Karstens et al, 2019, and Schneeberger et al., 2019, we prepared a panel of standard dilutions of a microbial community standard which expanded a large range of biomass, and processed to DNA extraction to sequencing along wit the BALFs specimens. These controls enabled us to define the lowest decontaminable biomass amount, validate the efficiency of the first decontamination with decontam, and exclude BALF specimens which biomass was too low to confidently decontaminate, and ii) to define a minimum abundance threshold to detect, and exclude spurious ASVs (likely cross-sample contaminants) in a sample-wise manner.

Furthermore, we did not apply shallow metagenomics sequencing, and preferred performing metagenomics on a subset of samples with sufficient biomass to avoid inclusion of spurious hits in the sequencings results.

Davis NM, Proctor DM, Holmes SP, et al. Simple statistical identification and removal of contaminant sequences in marker-gene and metagenomics data. Microbiome 2018; : 6(1):226.

Karstens L, Asquith M, Davin S, et al. Controlling for Contaminants in Low-Biomass 16S rRNA Gene Sequencing Experiments. mSystems 2019; : 4(4):e00290-19.

Schneeberger PHH, Prescod J, Levy L, et al. Microbiota analysis optimization for human bronchoalveolar lavage fluid. Microbiome 2019; : 7(1):141.

Providing additional details on how negative controls were used to differentiate true biological signals from possible contaminants—or discussing any limitations in this regard—would reinforce confidence in the observed differences. Given that Mycobacterium was detected at low frequency despite being the primary pathogen, addressing whether the observed Serratia/Yersiniaceae abundances could have been affected by technical or environmental factors would add further clarity.

As mentioned in the above response, we provide extensive details on the strict decontamination strategy we applied. We do indeed discuss a limitation of our decontamination scheme in the section strength and limitations lines 330-337: we could not collect sampling negative controls such as saline, oral and bronchoscope washes which we serve as negative controls for the sampling phase.

Of note, in an independent, subsequent pilot BALF procedure in the same clinic (but not included in this study), we collected bronchoscope wash and mouth rinse as sampling control, along with a BALF specimen, and generated microbiome sequencing data. Both bronchoscope wash and mouth rinse showed no traces of *Serratia* nor *Yersiniaceae, while Serratia* dominated the BALF specimen, suggesting that systematic contamination during sample collection or processing was unlikely.

We indeed detected *Mycobacterium* in only one NTM patient. Previous studies have also reported the same result (Caverly et al., 2016; Sulaiman et al., 2018, Hu et al., 2020) indicating the detection limits of 16S rRNA amplicon sequencing. Though *Mycobacterium* was the primary pathogen, biomass does influence its detection level in the 16S rRNA amplicon sequencing data.

Of note, when we applied targeted *Mycobacterium* high throughput sequencing with specific *Mycobacterium*-primers (deeplex, Genoscreen) on a TB patient (MSO-60), *Mycobacterium* was detected, and resistance profiles were defined on this BALF specimen:

**Figure 1**. **DEEPLEX® Myc-TB Report (GenoScreen) of TB patient MSO-60**. Drugs abbreviations: RIF: Rifampicin, INH: Isoniazid, PZA: Pyrazinamide, EMB: Ethambutol, SM: Streptomycin, FQ: Fluoroquinolones, KAN: Kanamycin, AMI: Amikacin, CAP: Capreomycin, ETH: Ethionamide, LIN: Linezolid, BDQ: Bedaquiline, CFZ: Clofazimine. 1: resistant, 0: susceptible.

Overall, based on i) our rigorous decontamination strategy, ii) previous studies reporting high dominance of *Serratia* in TB patients prior to anti-TB therapy, iii) the specificity of microbiome structure in TB patients given that the specimens were collected over a period of 14 years we conclude that the *Serratia* traits are very unlikely to be environmental noise/contamination.

Hu Y, Kang Y, Liu X, et al. Distinct lung microbial community states in patients with pulmonary tuberculosis. 2020; : 63(10):1522-1533.

Caverly LJ, Carmody LA, Haig SJ, et al. Culture-Independent Identification of Nontuberculous Mycobacteria in Cystic Fibrosis Respiratory Samples. PLoS One 2016; : 11(4):e0153876.

Sulaiman I, Wu BG, Li Y, et al. Evaluation of the airway microbiome in nontuberculous mycobacteria disease. Eur Respir J 2018; : 52(4):1800810.

2.2 Clarifying the Functional and Pathogenic Role of Serratia in TB
The identification of Serratia species in TB and NTM-LD patients is an interesting finding, and expanding the discussion on its potential biological role could greatly enrich the manuscript. Since different Serratia species exhibit diverse functional properties, ranging from commensalism to opportunistic pathogenicity, further elaboration on possible mechanisms of interaction with Mycobacterium or host immune responses would enhance the interpretation of these findings.

Thank you for your comment. To elaborate on the possible mechanisms of interactions between *Serratia sp.* and *Mycobacterium,* we discussed the novel findings of Eze and colleagues who demonstrated *Serratia marcescens* promoted the growth of *Mycobacterium tuberculosis* in an *in vitro* assay. The authors showed that specific molecules secreted by *Serratia* namely siderophores were used by *Mycobacterium tuberculosis* to extract iron from the environment necessary to its growth: Lines 315-319 (Discussion).

To strengthen the study’s conclusions, the authors might consider:

- Discussing whether Serratia enrichment in TB patients could be linked to immune modulation, microbial competition, or metabolic interactions within the lung microbiome.

This in a good point, thank you.

*Serratia*´s infection of the lung occurs mainly in immunocompromised patients. Previous studies based on *Serratia* *marcescens*- the most studied *Serratia* species- revealed the mechanisms used by *Serratia* *marcescens* to evade the immune system namely i) impairment of cellular immunity via production of serralysin metalloprotease (Kida al, 2006); ii) suppression of paralytic peptide (PP) activation via hemocyte killing (Ishii et al., 2012) and iii) the PhoP/PhoQ system Barchiesi et al., 2012.

Some of these mechanisms, and immune cells are shared with the immune routes, and interactions between *Mycobacterium tuberculosis* and the immune system such as apoptosis. Thus, a possible mechanism might be that when *Mycobacterium sp.* overwhelms the immune system during the onset of the infection, *Serratia* *sp.* opportunistically escapes the immune response, and overgrowths. Subsequently, *Serratia sp.* overgrowth results in the availability of secondary metabolites called siderophores in the lungs milieu which support the growth *Mycobacterium sp.*

Whether metabolic interactions also play a role in *Serratia* enrichment in TB requires future investigations.

This paragraph is now added the discussion section lines 280-289 for immune interactions, and metabolic interactions 300-302, and lines 315-319 for microbial interactions

Kida Y, Inoue H, Shimizu T, Kuwano K. Serratia marcescens serralysin induces inflammatory responses through protease-activated receptor 2. Infect Immun. 2007 Jan;75(1):164-74. doi: 10.1128/IAI.01239-06. Epub 2006 Oct 16. PMID: 17043106; PMCID: PMC1828393.

Ishii K, Adachi T, Imamura K, Takano S, Usui K, Suzuki K, Hamamoto H, Watanabe T, Sekimizu K. Serratia marcescens induces apoptotic cell death in host immune cells via a lipopolysaccharide- and flagella-dependent mechanism. J Biol Chem. 2012 Oct 19;287(43):36582-92. doi: 10.1074/jbc.M112.399667. Epub 2012 Aug 2. PMID: 22859304; PMCID: PMC3476324.

Barchiesi J, Castelli ME, Di Venanzio G, Colombo MI, García Véscovi E. The PhoP/PhoQ system and its role in Serratia marcescens pathogenesis. J Bacteriol. 2012 Jun;194(11):2949-61. doi: 10.1128/JB.06820-11. Epub 2012 Mar 30. PMID: 22467788; PMCID: PMC3370626.

- Exploring alternative explanations for the presence of Serratia, such as potential environmental exposure or prior antibiotic use.

This a good point. Thank you.

Environmental exposure: Given the diverse lung pathologies where *Serratia* was reported to be enriched in patient lungs prior to treatment, and the absence of *Serratia* in healthy lungs, this suggests *Serratia* is more likely be to an opportunistic infection rather than due to environmental exposure.

Prior antibiotic use: Hu et al, reported that abundance of *Serratia* in TB patients deceased after MTB negative conversion indicating anti-TB therapy impacted *Serratia* growth. Besides, prior antibiotic use, also targeting further organs such the gut could impact the lung inflammatory homeostasis, and lead to *Serratia* growth in the lungs. To our knowledge, such investigations are still to be conducted.

- Acknowledging that functional analyses (e.g., metagenomics, transcriptomics) would be needed to establish Serratia’s role in disease progression, and highlighting this as a potential avenue for future research.

Good point, thank you. Indeed, future investigations in addition to metagenomics to explore the functional repertoire of the lung microbiome should include transcriptomics, and metabolomics to decipher the precise expressed genes, and the metabolites involved in the disease.

We now added this paragraph in the discussion lines 308-319.

3. Discussion of Prior Literature and Knowledge Gaps

The manuscript would benefit from further contextualization within the broader field of TB microbiome research. A clearer discussion of how this study builds upon prior findings or what knowledge gap it addresses would help position it more effectively within the existing literature. It would be better to clarify (as early as the Introduction) whether this is the first study of the TB lung microbiome using BALF or whether previous studies have already explored similar microbial shifts. Several other studies have characterized TB-associated microbiome changes—yet the discussion does not compare the current findings to those results.

Thank you for your suggestion. We added the following aims to the introduction to emphasize the knowledge gap our study addressed:

i) Unlike previous studies which defined the lung microbiome in TB, and NTM-LD in distinct patient cohorts, our study directly contrasted the microbiome composition between TB, and NTM-LD patients in the same cohort.
ii) Besides defining the disparities in the diversity, and structure of the lung microbiome across TB, and NTM-LD, we performed indicator analysis to identify specific taxa which distinguish the two patient groups.

These aims are now added to the introduction lines 51-56.

We do discuss many studies which applied the same method (BALF + 16S rRNA amplicon sequencing) to generate lung microbiome data, and contrasted our results to these studies’ results. Indeed, from lines 234-264 we directly contrasted our results to previous studies including i) *Serratia* dominating the lungs in several pathologies, and in TB; ii) the challenge in detecting *Mycobacterium* using 16S rRNA amplicon sequencing; iii) and higher richness in TB patients.

Additionally, further exploration of whether Serratia has been previously reported in TB lung microbiomes (or any other lung condition) would strengthen the discussion by clarifying whether its presence is novel or aligns with existing knowledge. If Serratia’s role in the TB microbiome remains uncertain, acknowledging potential explanations—such as environmental factors, host immune interactions, or sample type differences—could provide a valuable perspective.

*Serratia* has been indeed reported in various lung conditions which we cited, and discussed in the manuscript, lines 239-252. In TB patients, Hu et al., 2020 [32] reported the dominance of distinct *Serratia* OTUs in TB patients.

The role of *Serratia* in TB is still to be fully investigated, and clarified. We discussed the potential growth promoting effect of *Serratia* on *Mycobacterium* based on recent findings, lines 315-319. Also, we added now further potential explanations of the role of *Serratia* in TB: see above response, now in the manuscript lines 280-289.

Finally, placing these findings within the broader landscape of respiratory microbiome research, including comparisons to conditions like pneumonia or chronic obstructive pulmonary disease (COPD), would offer deeper insights into whether the observed microbiome shifts are specific to TB or part of a more general lung disease signature

Thank you for your comment. Indeed, genus *Serratia* has been reported to dominate the lung microbiome of pneumonia, COPD patients as we reported in the manuscript lines 241-246.

Previous studies [21-31] reported that *Serratia* dominated or increased in various lung pathologies, especially during exacerbated/active disease phase. Hu et al., 2020 found distinct *Serratia* OTUs strongly dominated TB patients prior to therapy, along this line, our results showed dominance of the genus *Serratia* across TB, and NTM patients; yet the sub-genus diversity of *Serratia* traits was significantly distinct across the two patient groups, with certain *Serratia* ASVs only associated to TB patients. Thus, we argue that the dominance/increase of *Serratia* *sp.* is shared among various lung pathologies more likely in the acute phase of the disease, while it is likely that sub-genus diversity of *Serratia* traits which colonize the lungs is partially disease-specific.

We now added the following paragraph to the manuscript discussion lines 268-279, and 294-302.

Expanding the discussion to explore potential mechanisms by which microbiome changes might influence TB disease progression would significantly enhance the impact of this study.

Thank you for your suggestion. We now added the following paragraph to the discussion lines 303-309.

Mechanisms by which microbiome changes influence TB disease onset, and progression remain poorly understood. Pioneer study Zhang et al, 2022 [37] revealed distinct lung microbial functions in active TB patients including depleted metabolic functions involved in the biosynthesis of lipid metabolism, and increased metabolism of terpenoids and polyketides. Besides, Zhou et al., 2015 examined lung biopsies of TB patients, and found *Porphyromonas* associated with TB lesions suggesting it might be a co-factor for lesions formation.

Zhou Y, Lin F, Cui Z, Zhang X, Hu C, Shen T, Chen C, Zhang X, Guo X. Correlation between Either Cupriavidus or Porphyromonas and Primary Pulmonary Tuberculosis Found by Analysing the Microbiota in Patients' Bronchoalveolar Lavage Fluid. PLoS One. 2015 May 22;10(5):e0124194. doi: 10.1371/journal.pone.0124194. PMID: 26000957; PMCID: PMC4441454.

Integrating insights into host-microbe interactions, immune responses, or microbial competition within the lung environment could provide a more comprehensive interpretation of the findings.

This is indeed an import aspect. Following our above responses, we now address the potential role of the host immune responses: i) immune components/cells may be overwhelmed by *Mycobacterium* infection, giving an opportunity to Serratia to overgrowth in the lungs in the scenario of Serratia being an opportunistic pathogen in TB, and NTM infections; ii) *Serratia*-*Mycobacterium* interactions whereby *Serratia* supports the growth of *Mycobacterium tuberculosis* through iron uptake mechanisms.

This discussion points are now in lines 280-289, and 312-319.

Additionally, incorporating established concepts such as colonization resistance, dysbiosis, or microbial metabolic interactions would help frame the observed microbiome shifts within well-recognized biological frameworks. This could strengthen the discussion by offering potential explanations for why specific taxa, like Serratia and Yersiniaceae, may differ between patient groups.

Good point, thank you. We do discuss colonization resistance as a potential explanation for the higher diversity in TB patients (lines 265-267). In addition, we discuss other parameters as potential drives of the distinct lung microbiome within and between patient groups including prior medical history, the exact infectious strain(s), duration of the infection, physiopathology of the lungs, damaged gut-lung barriers to explain the heterogeneity in the structure, and diversity of *Serratia* traits lines 303-309.

4. Technical Clarifications

4.1 Data Availability
The raw sequencing data and processing scripts are provided; however, limited access to clinical metadata may constrain full reproducibility and comprehensive interpretation.

All the metadata used to generate the data of this manuscript are provided in Supplementary Table 2

4.2 Terminology
Since 16S rRNA sequencing is primarily used for bacterial taxonomic profiling, it would be helpful to clarify whether the metagenomic analysis refers to true whole-metagenome sequencing (WMS) or an extended 16S-based bacterial "metagenome" approach. Ensuring accurate terminology would improve the precision of the methods section and the interpretation of the results.

Our metagenomic analyses refer to true whole-metagenome sequencing (WMS) as mentioned in the manuscript lines 13-14, and extensively described in the supplementary material lines 218-235.

**Reviewer #2**: In this manuscript, the authors have studied retrospective samples of the lung microbiome of TB, NTM and non-infectious inflammatory disease patients using 16S rRNA amplicon sequencing as well as Whole Metagenome Sequencing. They have found that while these samples are primarily dominated by unclassified Yersiniaceae and Serratia with similar abundance across the disease groups, the sub-genus level analysis indicates difference in abundance certain strains across the groups as well as significant association of certain strains (ASV_7, ASV_21) with the disease state. Overall, the manuscript seems sound and requires minor corrections.

1. Given that the samples were collected retroactively, is it possible that the Serratia and unclassified Yersiniaceae strains evolved over time, especially for TB, as the disease progressed? The manuscript does not indicate when the TB was detected in the patient and how long the patient had it before sampling. The evolution and changes to the underlying strains might be interesting to investigate.

Thank you for this great point. We do agree that investigating the evolution of lung microbiome from the start of the infection/active disease is crucial to fully understand the role of the microbiome in the progression of the disease. For this study, we used BALF specimens collected to define the diagnosis. The patients were referred to our clinic with the suspicion of the disease to establish the final diagnosis, and the personalized therapy. The patients did fill-in a questionnaire regarding their current, and previous medial history. However, we could not determine the exact time point when the patient got infected, nor when the disease became active.

We acknowledge that the duration of infection, active disease very likely influences the lung physiology, and the microbiome structure, and contribute in explaining the observed difference in microbiome structure discussion lines 299-302, and limitations paragraph lines 344-347.

2. What is the corrected p value threshold for indicator taxa analysis? The corrected p-values have been provided but a threshold has not been given, that indicates significance of the analysis.

Thank you for your comment. For the corrected p values, we applied these thresholds: i) significance p ≤ 0.05; marginal significance 0.05 < p ≤ 0.1. We now indicate these thresholds in the supplementary material lines 200-202.

3. The quality of figures in the manuscript is low; however, once downloaded, the resolution is much better. Indeed, this is due to the high resolution required from journal.

4. There are minor grammatical errors in the supplemental section of the manuscript:
a) Line 123: "calculated based": Thank you, we now completed the sentence to “based on the resulting alignment”.

b) Line 133: "which null hypothesis is" Thank you, we corrected it to “of which null hypothesis is”
c) Line 164: "if a within a given sample". Thank you, we corrected to “if within a sample”
d) Line 177: "which DNA concentration" Thank you, we corrected it to “with DNA concentration”
e) Line 178: grammatical structure of the sentence needs to be corrected. Thank you, we now re-structured the sentence to “After excluding these BALF samples, n=123 adjusted ASVs remained in the dataset.”
f) Line 183: "randomly 10 000 reads". Thank you, we now corrected the sentence to “randomly drew 10 000 reads”.
g) Line 189: "which pairwise distance" Thank you. We corrected the sentence to “sequences which harbored pairwise”

5. What database was used for taxonomic assignment for whole-metagenome sequencing? This has not been mentioned in the supplemental section. We used Metaphlan4 to assign taxonomy to the whole-metagenome sequencing as mentioned in line 232.

6. Lines 246 and 249 in the supplemental section - has corrected p-value been used for Kruskal Wallis test? Unlike lines 251 and 255, this has not been mentioned/clarified.

This a good point, thank you. Indeed, in lines 246-249 we applied the Kruskal-Wallis test to the main phyla, and genera. The resulted p values were all > 0.05, so we reported the differences as non-significant, and did not apply further correction for multiple testing. On the other hand, in lines 251-255, we applied correction for multiple testing to assess whether the significance of the first test holds significant after correcting for multiple testing.

**Reviewer #3**: The manuscript "Serratia sp. traits distinguish the lung microbiome of patients with tuberculosis (TB) and non-tuberculous mycobacterial (NTM) lung diseases" describes the composition of the lung microbiota of patients with tuberculous mycobacterial infections and non-tuberculous lung disease. The authors aim to define the compositions prio to the initiation of therapy but also to unravel the role of the microbiome as the initiator or progessor of the development of mycobacterial lower respiratory infections.

The study is technically well performed but lacks some in-depth interpretation of the data. The authors use the data of twoo patients groups only: TB vs NTM lung disease. As the authors mention at page 13, the study lacks a group of healthy controls although it is clear to obtain such samples beacause of ethical reasons. However, it would be worthwhile to present the data of the four non-infectious patients. Sure, the statistical value would be very low due to the low number of non-infectious inflammatory disease, but might underline the typical presence of Serratia and (unclassified) Yersiniaceae in the other groups.

Thank you for your suggestion. We do actually present the data of the four non-infectious patients, and provide the taxonomy composition at the different resolution levels presented in supplementary Figures S1, and S2. In addition, we compared the abundances of the main phyla, and genera across TB, NTM-LD, and the non-infectious patients, and found similar abundances of the major phyla, and of the genera “unclassified Yersiniaceae” and *Serratia*. These results are presented in the supplementary material lines 243-256.

Another question is raised by the fact that some individuals do not harbor any Serratia and/or (unclassified) Yersiniaceae but are colonized/infected by Acinetobacter or Proteus. Which underlying mechanism is superior to Serratia or (unclassified) Yersiniaceae that might give the an ecological advantage that is probably present in some of the Acinetobacter of Proteus spp.?

Thank you, this is indeed a great point.

There is still not enough investigation on co-infections of *Mycobacterium sp*. and further bacterial pathogens, and even less data on the dynamics of these co-infections. It had been reported that *Acinetobacter baumannii* is the second more frequent co-infection in TB after *Pseudomonas aeruginosa* (Bir et al., 2024).

Besides, we learnt from Gupta et la., 2022, and others that *Serratia* dominated in exacerbated COPD, interstitial lung diseases and sarcoidosis, but not the stable COPD patients. Knowing that the exacerbated COPD phase is characterized by increased airway inflammation, and mucus production, it is possible/likely that *Serratia* co-infection takes over later in the co-infection dynamics once the airways are more damaged by the original infection, and subsequent co-infections. Thus, parameters such as time/duration since the activation/infection of the disease, and inflammation score/severity of the airway will help clarify the co-infections sequence in *Mycobacterium sp.* infection.

We added now this point to the discussion lines 290-302.

Bir R, Ranjan R, Gunasekaran J, Chatterjee K, Karteeka D, Rai A, Gupta S, Karlapudi P, Joshi I, Gupta RM. Prevalence of Co-infection of Culture-Proven Bacterial Pathogens in Microbiologically Confirmed Pulmonary Tuberculosis Patients From a Tertiary Care Center. Cureus. 2024 Aug 8;16(8):e66482. doi: 10.7759/cureus.66482. PMID: 39247035; PMCID: PMC11380723.

Technically, the numbers of reads are high enoough to get the resolution to detect low abundance bacteria. Whether the V3-V4 region of the 16S rRNA-gene is sufficient is part of discussion, as it might result to the determination at genus- of subgenus level rather than at species level.

It is advisable to expand the vision of the authors to the issues raised above.

We agree that the amplicon-based profiling of the microbiome provides a low taxonomical resolution, reaching only genus level at best. We chose this method to use the advantage of the relatively lower microbial biomass required to generate the data, and which characterized the BALF specimens.

To fine-scale the taxonomical resolution, we applied Whole Genome Sequencing (WGS) method on a few BALF specimens only which harbored sufficient microbial biomass to generate reliable WGS data to explore the species level taxonomy of the lung microbiome within these samples.
